# Transcriptome profiling of laser-captured germ cells and functional characterization of *zbtb40* during 17alpha-methyltestosterone-induced spermatogenesis in orange-spotted grouper (*Epinephelus coioides*)

**DOI:** 10.1186/s12864-020-6477-4

**Published:** 2020-01-23

**Authors:** Xi Wu, Yang Yang, Chaoyue Zhong, Yin Guo, Shuisheng Li, Haoran Lin, Xiaochun Liu

**Affiliations:** 1 State Key Laboratory of Biocontrol, Guangdong Province Key Laboratory for Improved Variety Reproduction of Aquatic Economic Animals, Institute of Aquatic Economic Animals, School of Life Sciences, Sun Yat-sen University, Guangzhou, 510275 China; 2 Southern Laboratory of Ocean Science and Engineering, Zhuhai, 519000 People’s Republic of China

**Keywords:** Spermatogenesis, Laser capture microdissection, Transcriptome, *zbtb40*

## Abstract

**Background:**

Spermatogenesis is an intricate process regulated by a finely organized network. The orange-spotted grouper (*Epinephelus coioides*) is a protogynous hermaphroditic fish, but the regulatory mechanism of its spermatogenesis is not well-understood. In the present study, transcriptome sequencing of the male germ cells isolated from orange-spotted grouper was performed to explore the molecular mechanism underlying spermatogenesis.

**Results:**

In this study, the orange-spotted grouper was induced to change sex from female to male by 17alpha-methyltestosterone (MT) implantation. During the spermatogenesis, male germ cells (spermatogonia, spermatocytes, spermatids, and spermatozoa) were isolated by laser capture microdissection. Transcriptomic analysis for the isolated cells was performed. A total of 244,984,338 clean reads were generated from four cDNA libraries. Real-time PCR results of 13 genes related to sex differentiation and hormone metabolism indicated that transcriptome data are reliable. RNA-seq data showed that the female-related genes and genes involved in hormone metabolism were highly expressed in spermatogonia and spermatozoa, suggesting that these genes participate in the spermatogenesis. Interestingly, the expression of *zbtb* family genes showed significantly changes in the RNA-seq data, and their expression patterns were further examined during spermatogenesis. The analysis of cellular localization of *Eczbtb40* and the co-localization of *Eczbtb40* and *Eccyp17a1* in different gonadal stages suggested that *Eczbtb40* might interact with *Eccyp17a1* during spermatogenesis.

**Conclusions:**

Our study, for the first time, investigated the transcriptome of the male germ cells from orange-spotted grouper, and identified functional genes, GO terms, and KEGG pathways involved in spermatogenesis. Furthermore, *Eczbtb40* was first characterized and its role during spermatogenesis was predicted. These data will contribute to future studies on the molecular mechanism of spermatogenesis in teleosts.

## Background

In animals, spermatogenesis is a developmental process in which diploid male germ cells transform into haploid functional male gametes in a tight spatial and temporal organization. Spermatogonia develop into primary spermatocytes, and then primary spermatocytes are transformed into mature spermatozoa through two meiotic divisions. These processes are achieved by a complex interplay of genes and hormones [1]. During spermatogenesis, the testis contains different male germ cells and somatic cells. Revealing the transcriptome changes of these cells can facilitate our understanding of the mechanism of spermatogenesis in vertebrates. However, it is difficult to isolate the specific germ cells to investigate their transcriptome due to the complicated testis structure. Some approaches have already been used to quantify and localize unique genes in the testis during spermatogenesis, such as in situ hybridization [2, 3], immunohistochemistry, and serial analysis of gene expression [4, 5]. These methods can determine the spatial expression of specific gene during spermatogenesis, but cannot provide high throughput gene expression profiles in specific cell populations.

Laser capture microdissection (LCM) technology was developed in the late 1990s to obtaining targeted cell populations from tissue sections. This method was first used to study the gene expression in human cancer cells, and then widely used to study the spermatogenesis in rodents and fish species [6–8]. Although LCM can capture the targeted cells from the tissues, it is difficult to obtain enough RNA sample for traditional high-throughput RNA sequencing. In recent years, the single-cell RNA-sequencing technique has been developed, which only requires low amount of RNA to construct the transcriptome library. Therefore, high throughput gene expression analysis in specific germ cells during spermatogenesis can be performed by a combination of LCM and scRNA-seq.

Orange-spotted grouper is a protogynous hermaphroditic fish underlying sex change from female to male in its life history [9]. It has been considered as a good fish model for studying the sex reversal. As it is difficult to obtain the natural male fish, few studies on the spermatogenesis has been carried out in the orange-spotted grouper. In order to reveal the regulatory mechanism of spermatogenesis, in this study, LCM was applied to obtain the four germ cell types from the gonad of male orange-spotted grouper induced by MT implantation, including spermatogonia (SG), spermatocytes (SC), spermatids (ST), and spermatozoa (SZ), and the transcriptome sequencing was conducted subsequently. Through the transcriptomic analysis, *zbtb* (Zinc finger and BTB domain-containing protein) genes were found to be differentially expressed in different cell types. ZBTBs are an evolutionarily conserved family of transcription factors. Approximately 60 ZBTB proteins have been identified involving in diverse functions including development, differentiation, and oncogenesis [10–12]. In recent years, ZBTB16 has been found to play an essential role in spermatogenesis by controlling the self-renew and differentiation of spermatogonium [13–16]. Therefore, the *zbtb* family genes were further characterized and their expression was investigated during the process of spermatogenesis in orange-spotted grouper.

## Results

### Developmental stages of gonads during MT-induced spermatogenesis

**Fig. 1:**
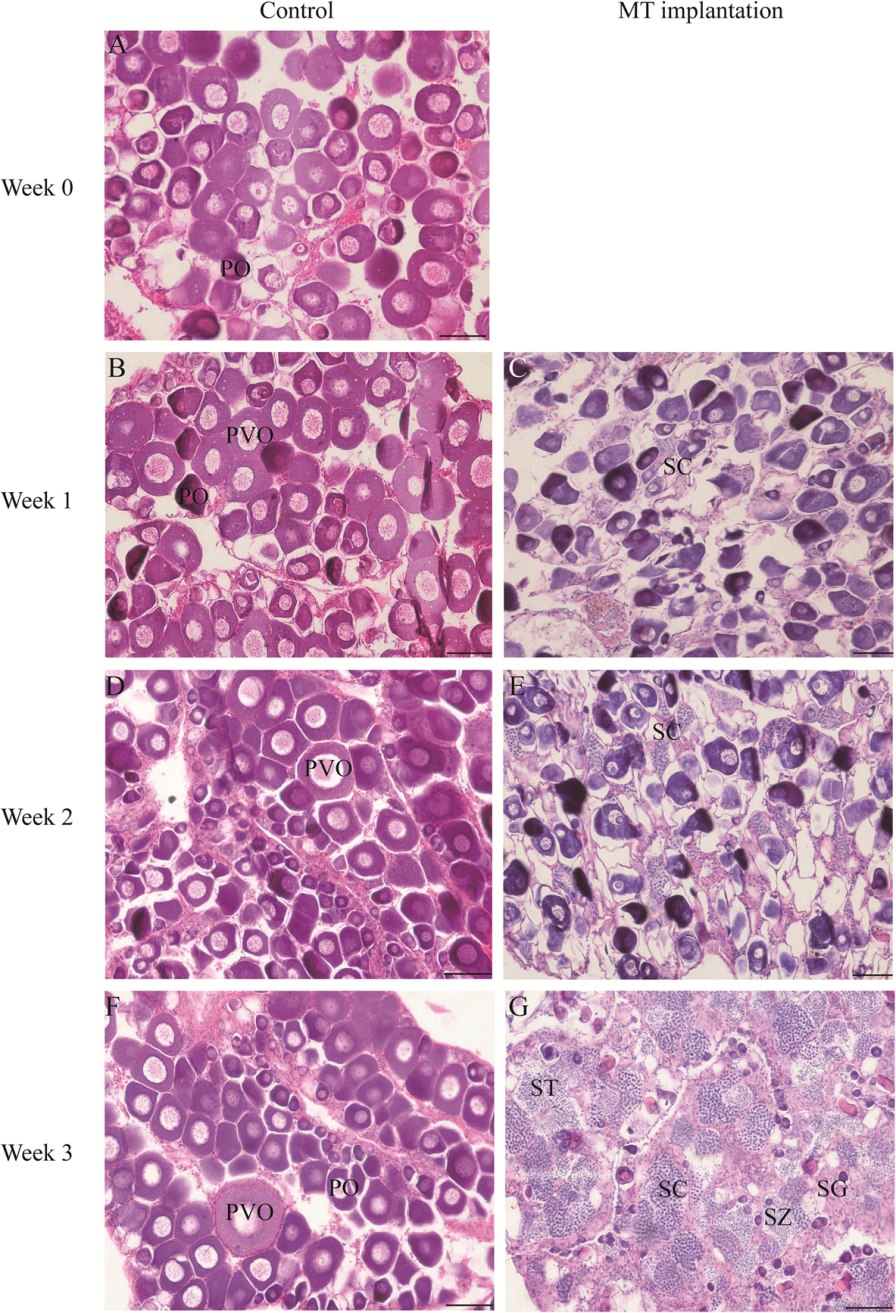
Gonadal histological morphology after MT treatment. **a, b, d and f** Histology of gonads in control fish. **c, e and g** Histology of gonads after MT implantation. PO, primary-growth stage oocyte; PVO, the cortical-alveolus stage oocyte; SG, spermatogonia; SC, spermatocyte; ST, spermatid; and SZ, spermatozoa. Scale bars = 50 um

Oocytes in the sham group remained in the primary-growth stage throughout the experimental period (Fig. 1a, b, d and f). In contrast, the fish in MT-implanted group underwent sex reversion from female to male. At the first week after MT implantation, the gonads were characterized by degeneration of oocytes and simultaneous proliferation of spermatogenic cysts (Fig. 1c). At the 2 weeks after MT implantation, the gonads entered into the intermediate transitional stage. Atretic oocytes were absorbed, and a mass of SC and ST were present (Fig. 1e). At the 3 weeks after MT implantation, the gonads entered into the late stage of spermatogenesis, and were occupied by SC and ST (Fig. 1g).

### Capture of the targeted cells

**Fig. 2:**
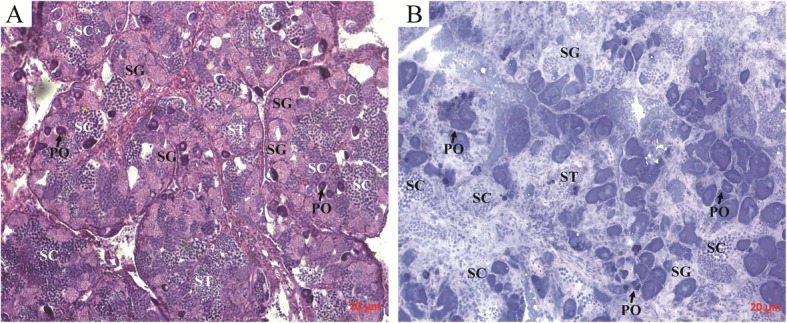
Gonadal structure by different staining methods. **a** Histological structure of gonad in MT treatment by H&E staining. **b** Histological structure of fresh gonad in MT treatment by quick staining. SG, spermatogonia; SC, spermatocyte; ST, spermatid; SZ, spermatozoa; and PO, primary-growth stage oocyte. Scale bars = 20 um

The morphology of SG, SC, ST, and SZ were characterized by hematoxylin and eosin (H&E, Fig. 2a) and quick staining (Fig. 2b). During spermatogenesis, the size of male germ cells decreases gradually, and the chromatin of the cells is condensed constantly [1]. Thus, different male germ cells can be distinguished by their morphology, size and density of chromatin [17]. In morphology, SG cell is irregular round, while SC, ST and SZ cells are more regular round. The size of four male cells become smaller gradually (SG > SC > ST > SZ). The staining of nuclei reflecting the density of chromatin is darker constantly in SG, SC, ST and SZ. Under the microscope, four different male germ cells were isolated by laser in PEN slices based on these characteristics. SG, SC and ST cells were captured from the gonad at the middle stage of spermatogenesis. SZ cells were obtained from the gonad at the late stage of spermatogenesis.

### Validation of the sample quality

**Fig. 3:**
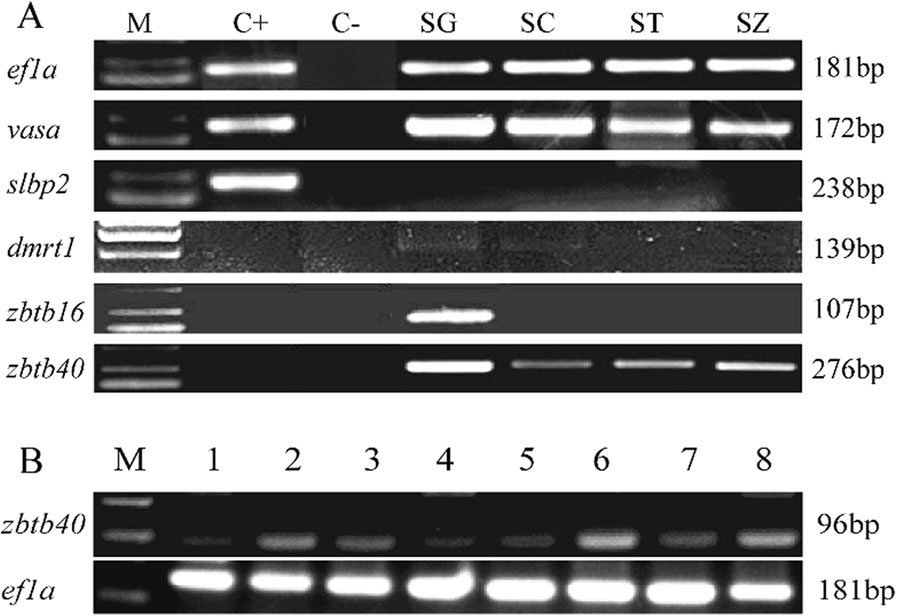
Expression of several sex-related genes in different tissues. **a** Expression of sex-related genes in four laser-captured cell types. The gene names were labeled on the left, and the length of genes was labeled on the right. M, marker 2000; C+, the cDNA of ovaries as a positive control; C-, template-free as a negative control; SG, spermatogonia; SC, spermatocyte; ST, spermatid; SZ, spermatozoa. **b** Tissue distribution of *zbtb40* in eight tissues. *Ef1a* was used as the reference gene. M, marker 2000; 1, the whole brain; 2, heart; 3, head kidney; 4, liver; 5, kidney; 6, pituitary; 7, ovary; 8, testis

Five genes were used to validate the quality of the LCM-derived RNA samples (Fig. 3a). *Ef1a* is a reference gene and commonly used as an internal control for gene expression analysis. Its expression was detected in all four types of male germ cells at similar levels. *Vasa* is a germ cell marker [18] whose expression was also found in the four cell types. *Slbp2* was specifically expressed in the oocyte of orange-spotted grouper [19], and no expression was detected in any male germ cells except for in the positive control (the gonad containing primary-growth stage oocytes). *Dmrt1* was specifically expressed in the spermatogenic cells of orange-spotted grouper [20]. Here, its expression was only detected in SG and SC. *Zbtb16* was expressed in spermatogonia specifically in orange-spotted grouper.

### Quality of the transcriptomic data

Four cDNA libraries (SG, SC, ST, and SZ) were constructed. A total of 253,040, 890 raw reads were obtained, and a total of 244,984,338 clean reads were produced after removing low-quality reads and adapter sequences (Table 1). The average Q20 and Q30 values were 95.74 and 90.57%, respectively. The GC content was 44.08–47.47%.

**Table 1:** Summary of the sequence assembly after transcriptome sequencing

Sample	Raw_reads	Clean_reads	Clean_bases (Gb)	Error_rate (%)	Q20 (%)	Q30 (%)	GC (%)
SG	61,624,022	60,415,044	8.98	0.02	96.34	91.33	45.81
SC	57,374,966	55,414,904	8.15	0.02	95.22	90.11	44.08
ST	63,794,266	61,512,408	9.1	0.02	95.65	90.45	47.47
SZ	70,247,636	67,641,982	10.05	0.02	95.75	90.4	47.17
Total	253,040,890	244,984,338	36.28	–	–	–	–

*SG* spermatogonium; *SC* spermatocyte; *ST* spermatid; *SZ* spermatozoa; *Gb* Giga base; *Q20* percentage of bases with a Phred value of at least 20; Q30, percentage of bases with a Phred value of at least 30

### Differentially expressed genes (DEGs) among the four cell types

Fragments per kilo base millions (FPKMs) were used to quantify the gene expression levels. The FPKM values of each gene in the four cell types were compared, respectively. There were 16,406 up-regulated genes and 11,054 down-regulated genes in SG compared to SC. A total of 15,845 up-regulated genes and 6895 down-regulated genes were identified in SC compared to ST. The ST had 8320 up-regulated genes and 15,797 down-regulated genes compared to SZ. Among the four cell types, 4483 DEGs were identified for further analysis.

### GO and KEGG enrichment of DEGs

**Fig. 4:**
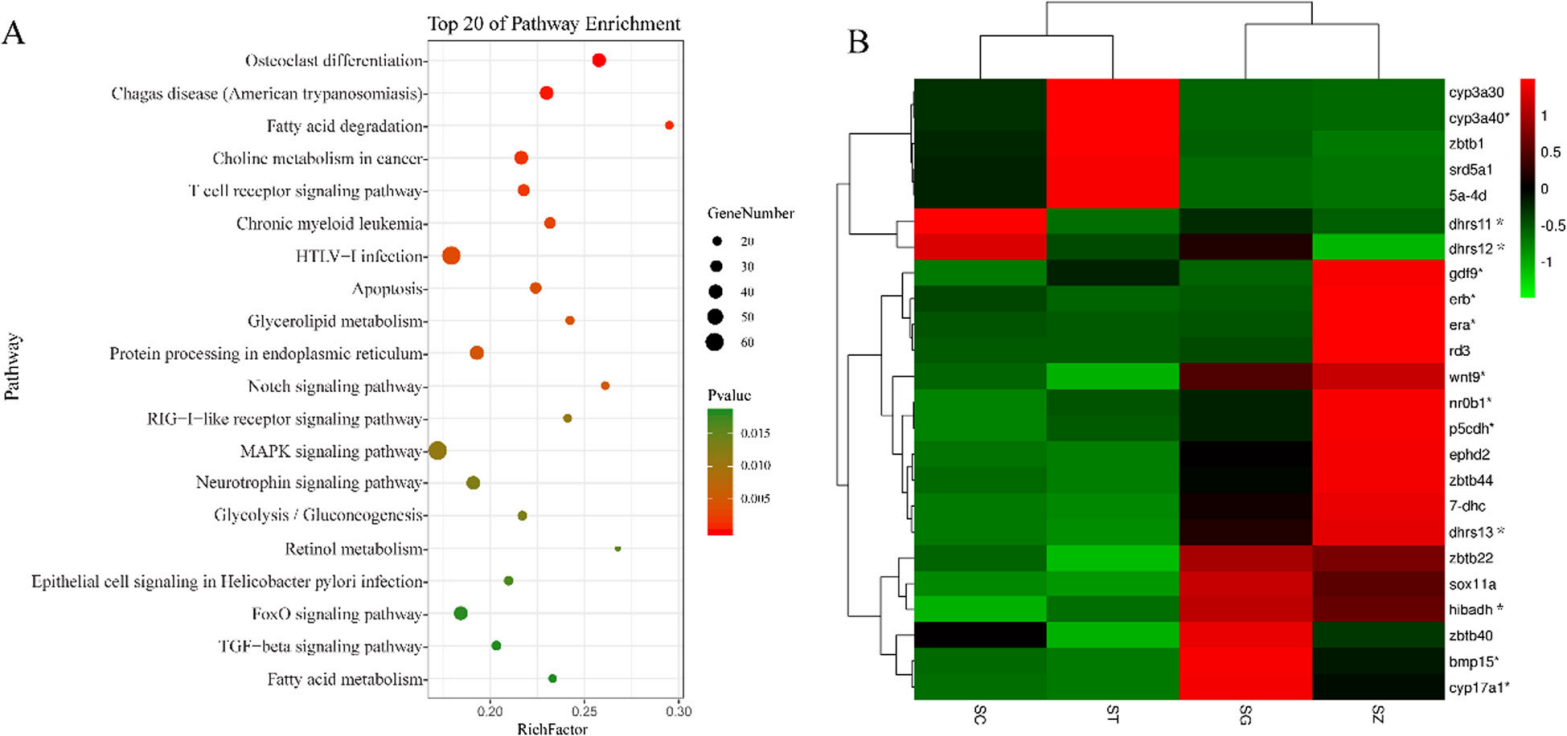
The enrichment and expression analysis among the 4483 DEGs. **a** The top 20 pathways of KEGG analysis with the significant difference. **b** The heatmap based on the selected genes. Red indicated high expression and green low expression. The gene names were labeled on the right, and the validated genes by real-time PCR were marked by asterisk after the gene names. SG, spermatogonia; SC, spermatocyte; ST, spermatid; SZ, spermatozoa

In the biological process and molecular function categories of the 4483 DEGs, cellular process (GO: 0009987) and binding (GO: 0005488) were the most enriched GO terms (Additional file 1: Figure S1). The top 20 pathways were listed from KEGG enrichment (Additional file 2: Table S1). Among them, apoptosis pathway, MAPK signaling pathway, and retinol metabolism pathway, were enriched during spermatogenesis (Fig. 4a).

### Expression of functional genes putatively associated with sex differentiation and hormone metabolism

**Fig. 5:**
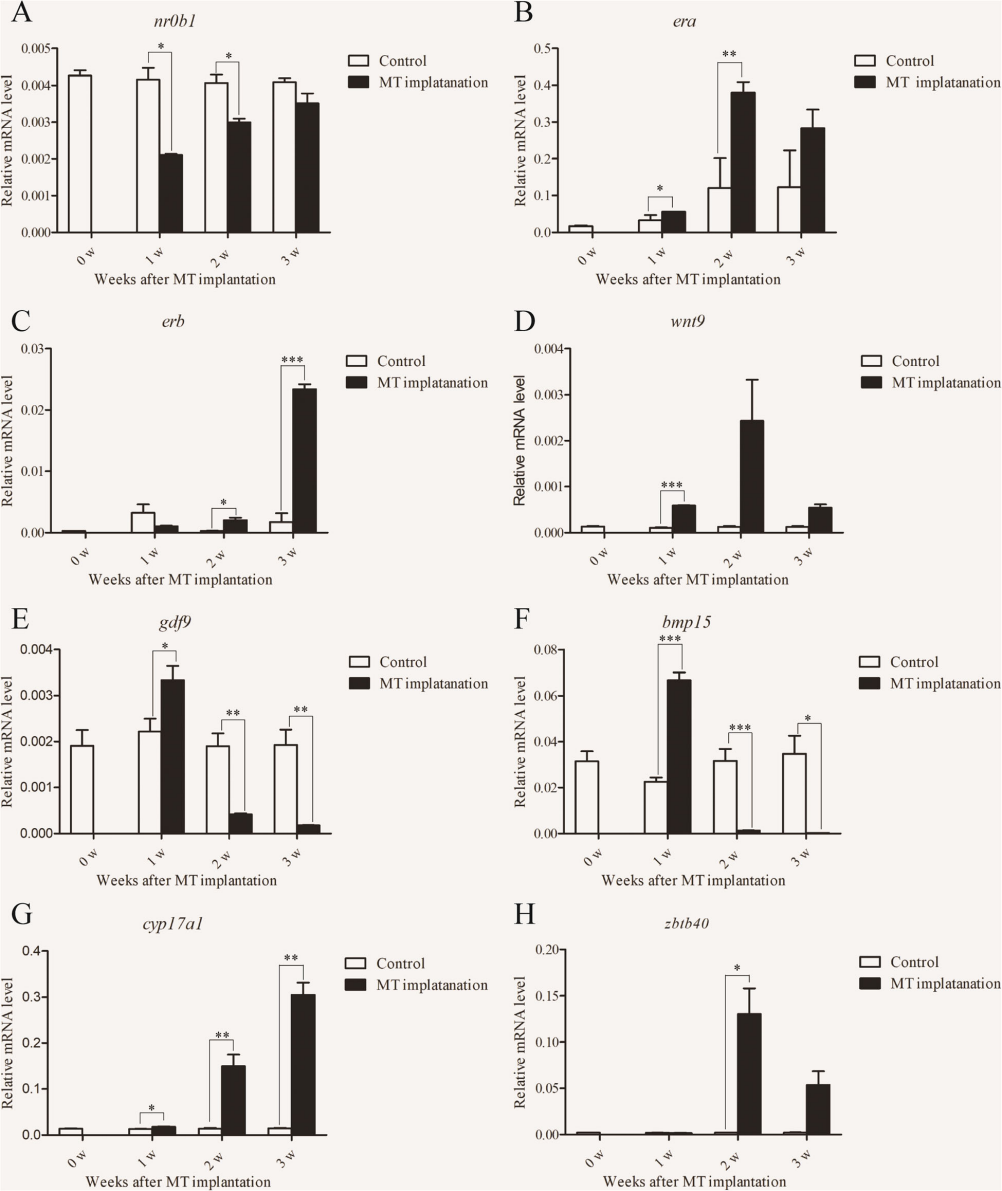
Validation of selected genes using real-time PCR during sex reversal. 0w, the female grouper before MT implantation; 1w, the early stage of sex reversal after 1week implantation; 2w, the middle stage of sex reversal after 2 weeks implantation; 3w, the late stage of sex reversal after 3 weeks MT implantation. The data was shown as mean ± SEM (n = 4 or 5) and the values with different asterisks were significantly different with a probability level <0.05 (P < 0.05). *Ef1a* was used as the reference gene. **a** *nr0b1*; **b** *erα;* **c** *erβ;* **d** *wnt9*; **e** *gdf9*; **f** *bmp15*; **g** *cyp17a1*; **h** *zbtb40*

From the transcriptomic data, the expression of 20 genes putatively related to sex differentiation and hormone metabolism were analyzed (Fig. 5 and Additional file 1: Figure S2). Eight sex differentiation genes, *sox11a* (SRY-Box Transcription Factor 11a), *nr0b1* (orphan nuclear receptor 0 B1), *erα* (estrogen reportor α), *erβ* (estrogen reportor *β*), *wnt9* (Wingless-type MMTV integration site family member 9), *gdf9* (oocyte-secreted growth differentiation factor 9), and *bmp15* (bone morphogenetic protein 15), and thirteen hormone-related genes, *cyp17a1* (steroid 17-alpha-hydroxylase/17, 20 lyase), *hibadh* (3-hydroxyisobutyrate dehydrogenase), *dhrs11* (dehydrogenase/reductase family member 11), *dhrs12* (dehydrogenase/reductase family member 12), *p5cdh* (delta-1-pyrroline-5-carboxylate dehydrogenase), *cyp3a40* (cytochrome P450 3A40-like), and *dhrs13* (dehydrogenase/reductase family member 13), which are involved in hormone metabolism were expressed in different cell types with FPKM values ranging from 2.62 to 604.7. Most of these genes were highly expressed in SG and SZ (Fig. 4b).

### Real-time PCR validation

Real-time PCR was performed to validate the expression patterns of 13 DEGs identified from the transcriptomic data. The expression of these genes was significantly changed during the spermatogenesis, which was largely consistent with their expression patterns in the transcriptomic data. Several female-related genes (*erα*, *erβ*, *gdf9*, and *bmp15*) increased significantly in the early stage of spermatogenesis (Fig. 5). The genes involved with hormone metabolism were also significantly increased during spermatogenesis (Additional file 1: Figure S2). These results indicate that the expressional analysis based on RNA-seq data was credible.

### Molecular cloning and sequence analysis of *zbtb40*

Among the 4438 DEGs in four male germ cells, we found that several *zbtb* genes (*zbtb1*, *zbtb22*, *zbtb40*, and *zbtb44)* were differentially expressed. ZBTB family proteins are transcription factors participating in various important functions. *Zbtb16* (*plzf*) was found to play an important role in the self-renewal and differentiation of the undifferentiated spermatogonia [13]. Whether the *zbtb* genes are involved in the regulation of spermatogenesis in orange-spotted grouper warrants further investigations. The expression of the *zbtb* genes (*zbtb1*, *zbtb22*, *zbtb40*, and *zbtb44*) was examined in spermatogenesis (Additional file 1: Figure S3). After further verification, the sequence and expression of *zbtb40* were further characterized.

**Fig. 6:**
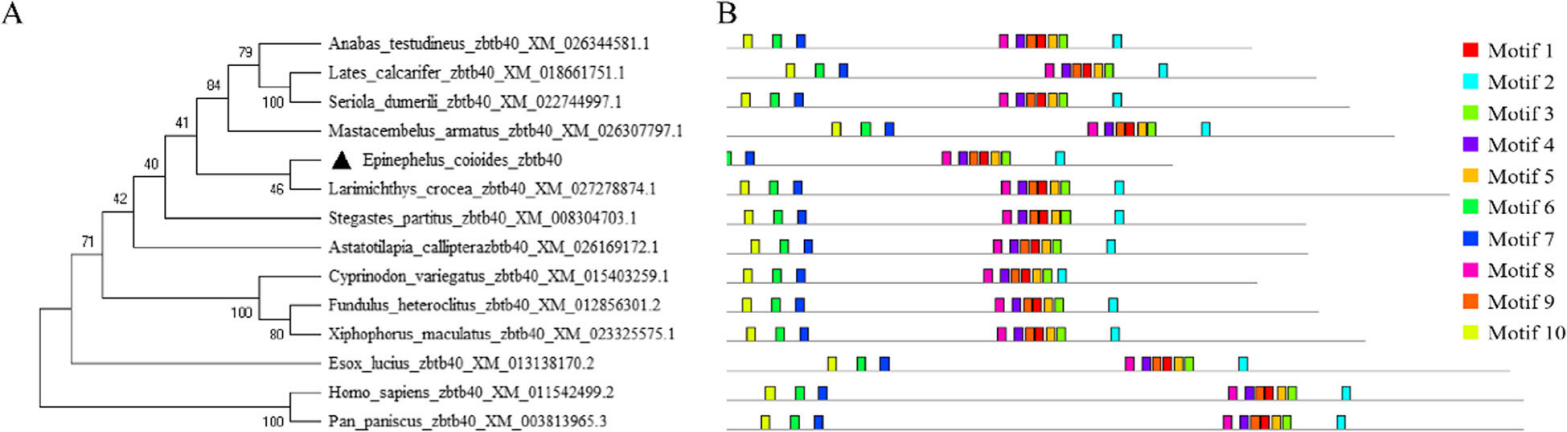
Phylogenetic relationships and conserved DNA motifs in *zbtb40* genes from 14 species. **a** Phylogenetic tree of *zbtb40*s were conducted by MEGAX using the neighbor-joining likelihood method with 1000 bootstrap replicates. Numerals at nodes were bootstrap values. The species names are followed by sequence accession numbers. **b** The distribution of conserved motifs in ZBTB40s. The motifs are displayed in different colored boxes. The sequence information for each motif is provided in Additional file 2: Table S2

The open reading frame (ORF) of *zbtb40* was cloned from the testis of orange-spotted grouper, and its sequence was submitted to GenBank (GenBank accession number, MN167853). The ORF of *zbtb40* is 2400 bp in length, encoding a protein of 799 amid acids (Additional file 1: Figure S4). A phylogenetic tree showed that *zbtb40* was clustered together with large yellow croaker (*Larimichys crocea zbtb40*) (Fig. 6a). The MEME web server was used to search the conserved motifs of *zbtb40s*. All *zbtb40s* were found to contain ten distinct conserved motifs (Fig. 6b). The DNA sequence of each motif site was displayed in Additional file 2: Table S2.

### The expression pattern of *zbtb40*

The mRNA expression of *zbtb40* in different tissues was examined by semi-quantitative PCR. The results revealed that *zbtb40* is highly expressed in the testis, heart and pituitary, with low expression in the brain, head kidney, liver and ovary (Fig. 3b).

The expression pattern of *zbtb40* during the MT-induced spermatogenesis was investigated by real-time PCR. The expression of *zbtb40* was hardly detected in the gonad of sham group and 1 week after MT implantation. In the gonad of 2 weeks after MT implantation, the expression of *zbtb40* was increased significantly compared to the sham group. However, the expression of *zbtb40* was decreased in the 3 weeks after MT implantation (Fig. 5h).

### In situ localization of *zbtb40* in gonads during spermatogenesis

**Fig. 7:**
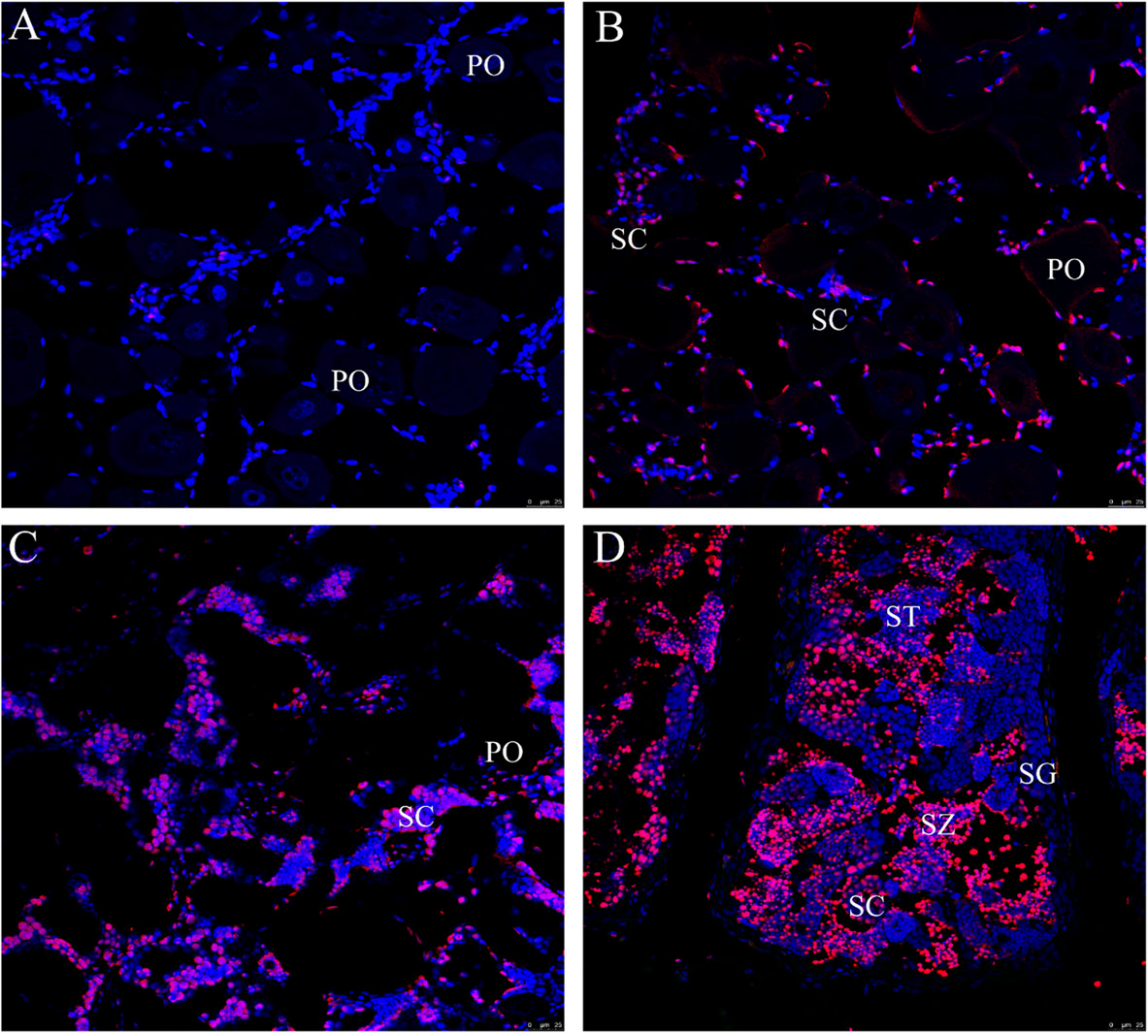
ISH analysis of *zbtb40* mRNA expression in the gonads during the sex reversal. **a** Gonads without MT treatment, **b** gonads after 1 week of MT implantation, **c** gonads after 2 weeks of MT implantation, **d** gonads after 3 weeks of MT implantation. Red signals indicate *zbtb40* and blue staining indicates nuclei. PO, primary-growth stage oocyte; ST, spermatids; SZ, spermatozoa. Scale bars = 50 μm

At the early stage of spermatogenesis, the *zbtb40* mRNA signals were not detected in the oocytes but were observed in the male germ cells and somatic cells (Fig. 7b)*.* At the middle stage of spermatogenesis, the *zbtb40* mRNA is mainly expressed in SG and SC (Fig. 7c). At the late stage of spermatogenesis, *zbtb40* was abundantly expressed in SG, SC, ST and SZ (Fig. 7d). In comparison, no *zbtb40* signal was detected in the ovary of the sham group (Fig. 7a).

### Co-localization of *zbtb40* and *cyp17a1* in ovary and testis

**Fig. 8:**
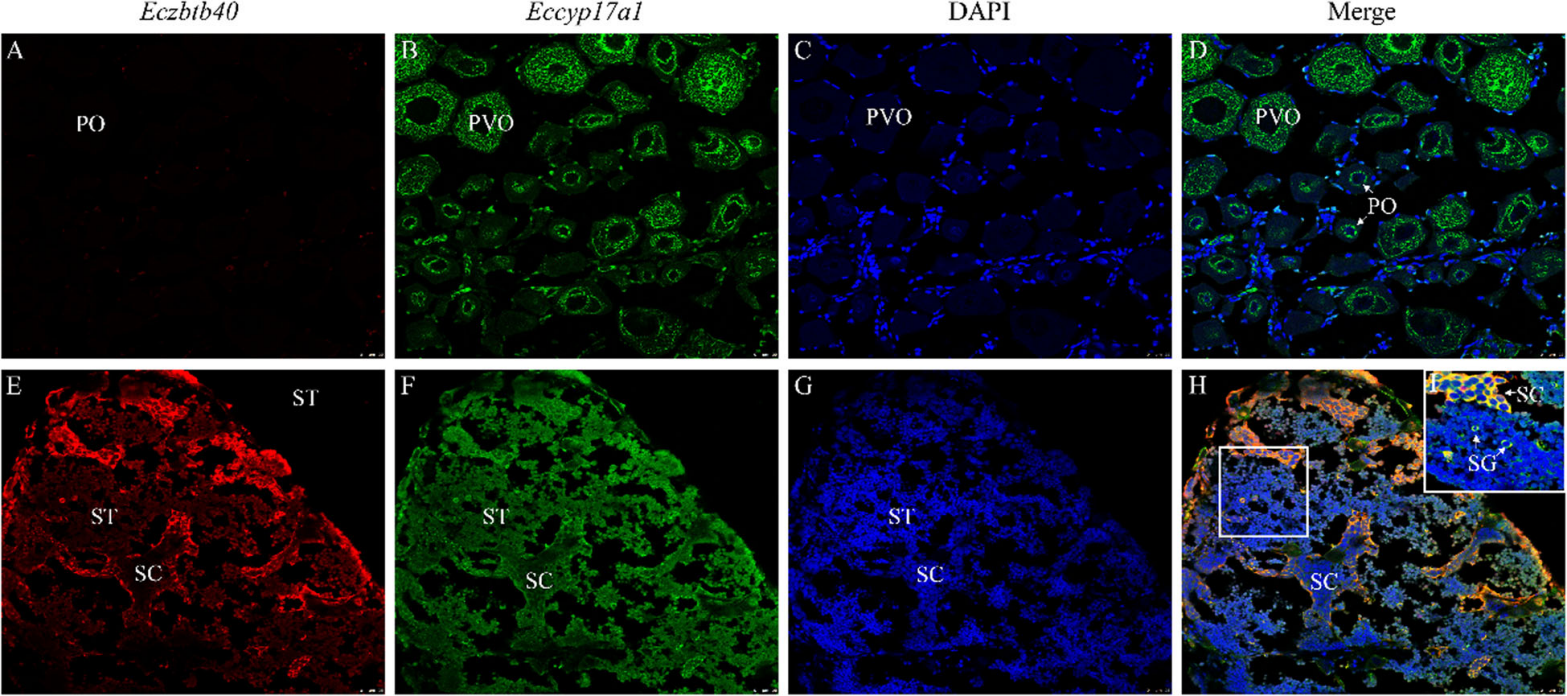
Co-localization of *zbtb40* and *cyp17a1* by FISH in the ovary (**a-d**) and testis induced by MT-implantation (**e-h**)*.* (**d**) The merge image of (**a-c**). (**h**) The merge image of (**e-g**). **i** The magnified image of (**h**). Gonadal sections were stained red for *zbtb40* mRNA, green for *cyp17a1* mRNA, blue for DAPI, and orange for merge signal. PO, primary-growth stage oocyte; PVO, the cortical-alveolus stage oocyte; SG, spermatogonia; SC, spermatocyte; ST, spermatid. Scale bars = 25 μm

Given the similar expression patterns of *zbtb40* and *cyp17a1* during spermatogenesis, the cellular co-localization of two genes in the gonad was examined in ovary and testis by fluorescence in situ hybridization (FISH, Fig. 8). In ovary, *cyp17a1* signals were clearly observed in the cytoplasm of oocytes where no *zbtb40* signal was found (Fig. 8a-d). *Cyp17a1* signals were located in the margin of oocyte, in the margin of nuclei in primary growth stage oocyte, and in the cytoplasm of cortical alveoli stage oocyte (Fig. 8b). In the gonad of the late stage of spermatogenesis, *zbtb40* signals were located in the cytoplasm of SG and SC, which were overlapped with the *cyp17a1* expression (Fig. 8e-h). Both *zbtb40* and *cyp17a1* had weaker expression in ST and SZ compared with SG and SC.

## Discussion

In the present study, transcriptome analysis was used to profile the process of spermatogenesis based on the LCM technology. These data enhance our understanding on the mechanism of spermatogenesis in orange-spotted grouper as well as other vertebrates.

The whole process of LCM must produce sufficient amount of RNA with high quality to ensure the reliability of the transcriptome results. In this study, we modified a previous protocol [21] to handle slides and tissues, samples staining and capture the cells for preserving the RNA integrity throughout microdissection. To verify the quality of the four targeted cells, several cell markers were detected. The similar expression levels of *ef1a* in different cells indicated the homogeneity of concentration in four cell types. *Vasa*, a germ cell marker [22], is required for proper germ cells development. Its expression levels were decreased gradually with the progress of spermatogenesis, which is consistent with the expression pattern of *vasa* in the male germ cells of gibel carp [22] and brown-marbled grouper [18]. As an oocyte-specific marker [19, 23], *slbp2* was used to distinguish the female germ cell and male germ cell. The non-expression of *slbp2* in four cell types indicated that targeted cells didn’t contaminate by female germ cells. *Dmrt1* was a spermatogenic cell marker [24] and is only expressed in SG and SC but not in ST and SZ in orange-spotted grouper. In zebrafish, *zbtb16* was proved as a spermatogonia marker [15], the expression of *zbtb16* demonstrated that the LCM-derived SG was spermatogonia. The expression patterns of these genes indicated the high quality of the captured cells.

At present, transcriptome resource for the spermatogenesis in orange-spotted groupers is rather limited. In this study, transcriptome analysis identified some pathways putatively involved in spermatogenesis, including apoptosis pathway, MAPK signaling pathway, and retinol metabolism pathway. These signal pathways were also reported in the spermatogenesis of Chinese mitten crab (*Eriocheir sinensis*) [25]. Among numerous DEGs, it is interesting to note that some traditionally female related genes were highly expressed in the gonad after MT implantation, such as *erα*, *erβ*, *gdf9* and *bmp15*. In mammals, estrogen receptors play crucial roles in spermatogenesis, e.g. the proliferation of gonocyte and spermatogonia, spermiation, sperm transport, and epididymal sperm maturation [26]. In eels, estrogen receptors are also expressed in the testis and are required for spermatogonial renewal [27, 28]. The high expression of estrogen receptors suggest that estrogen signals are also needed for spermatogenesis in grouper. GDF9 and BMP15, two members of the transforming growth factor beta family, were considered as female related genes promoting the oocyte maturation and folliculogenesis [29, 30]. However, several studies have reported the expression of *gdf9* and *bmp15* in testis, especially in male germ cells [31–33] and somatic cells [34]. The significantly increase of these genes indicated that female-related genes may also play important roles in spermatogenesis, providing a novel insight for understanding of the mechanism of spermatogenesis in groupers.

Some genes related to hormone metabolism were also differently expressed during spermatogenesis. The dehydrogenase/reductase family is short-chain superfamily, encoding various enzymes to participate in the metabolism of steroid hormones [35]. DHRS11 is a novel type of 17 *β*-hydroxysteroid dehydrogenase to tart part in the synthesis of estrogen and androgen in human [36]. In chicken, *DHRS12* has been identified for its plausible function in yolk and follicle developments [37]. CYP3A40, a cytochrome P450 member, hydroxylates testosterone to regulate the metabolism of hormone in medaka [38]. Our study showed that the expression of *dhrs11* and *cyp3a40* was significantly increased after MT implantation, while the expression of *dhrs12* was decreased during spermatogenesis. The specific expression patterns of the *dhrs11*, *dhrs12*, and *cyp3a40* genes may be important for hormone metabolism during spermatogenesis of groupers.

Few studies have examined the function of ZBTB40. We found that *zbtb40* is only expressed in male germ cells. Gender-specific expression of *zbtb40* indicated that it may participate in the process of spermatogenesis. FISH analysis showed that *zbtb40* was co-localized with *cyp17a1* in SG and SC during spermatogenesis. *Cyp17a1* is a 17*α*-hydroxylase and 17, 20-lyase to synthesize androgens and estrogens in steroid biosynthesis pathway [39]. In Cyp17-deficient mice, the male are infertile, and the androgen levels dropped significantly [40]. Similarly, in *cyp17a1*-deficient zebrafish, the androgen levels declined significantly, and their male-typical mating behaviors and secondary sex characters were compromised [41, 42]. The colocalization analysis indicated that *zbtb40* and *cyp17a1* may have interaction during the spermatogenesis in the orange-spotted grouper. However, the biological function of *zbtb40* during spermatogenesis requires further analysis.

## Conclusions

In conclusion, during the spermatogenesis, four different male germ cells were isolated by an optimized protocol of LCM and transcriptome analysis was performed. The RNA-seq data identified the signal pathways related to spermatogenesis, and revealed that the female-related genes and hormone metabolism-related genes participate in the process of spermatogenesis. The specific expression of *zbtb40* in male germ cells is highly suggestive of its role in spermatogenesis. Our study provides valuable data for understanding the regulatory mechanism of spermatogenesis in orange-spotted grouper and other protogynous hermaphroditic species.

## Methods

### Animals

Orange-spotted groupers were obtained from Guangdong Daya Bay Fishery Development Center (Huizhou 516,081, Guangdong, China). The fish were kept in indoor pools under controlled water temperatures of 22.7~27.8 °C. All fish were anesthetized with MS222 and then were sacrificed. All animal experiments were conducted in accordance with the guidelines and approval of the respective Animal Research and Ethics Committees of Sun Yat-Sen University.

### MT-induced spermatogenesis

In this study, the spermatogenesis was induced by MT (Sigma, USA) treatment artificially. The fabrication of the slow-release strips and MT implantation were referred to our previous paper with minor modification [43]. Fish (body weight, 1.90 ± 0.65 kg; body length, 43.75 ± 9.25 cm) were divided into two groups, sham group (*n* = 15) and MT implantation group (*n* = 15). The dosage of MT was 10 mg/kg body weight. Before implantation (Week 0), gonadal tissues of five fish were collected randomly. After MT implantation, five fish were sampled randomly every week from two groups, respectively. The experiment last for 3 weeks. For each fish, one piece of gonadal tissue was fixed in Bouin’s solution for histological examination, one piece of the gonad was immobilized by 4% paraformaldehyde for FISH, and the other piece of gonadal tissue was embedded with optimal cutting temperature compound (Sakura, USA) then frozen immediately in liquid nitrogen for LCM. All the other tissues were frozen immediately in liquid nitrogen, and then stored at − 80 °C until further use.

### Histology analysis

Gonadal tissues were embedded in paraffin after being fixed for 24 h in Bouin’s solution. The embedded blocks were sectioned at 5~6 μm and stained with H&E staining. The gonadal sections were classified by light microscopy (Nikon, Japan).

### Cryostat sections of gonad for LCM

The RNase-free Membrane Slides (MMI, Switzerland) were used to mount the cryosections. A series of procedures were produced before sectioning. The slides were incubated in super clean bench under ultraviolet (UV) radiation for 30 min. Then the slides were coated with 0.1 mg/ml poly-L-lysine (Sigma, USA) for 5 min, and rinsed by 0.1% DEPC (Sigma, USA, Diethyl pyrophosphate). At last, the slides were dried and stored in a sealed box for further use [44].

Before sectioning, the microtome (Leica, Germany) was wiped down with RNase inhibitor (Ambion, USA) to avoid cross-contamination, and a new blade (Lecia, Germany) treated with RNase inhibitor was used to cut each sample. The gonad blocks were put into Leica Microtomes for 30 min to adjust the sectioning temperature (-20 °C~-25 °C). The testis was cryosectioned at 6 μm.

### Quick staining

After drying for 3 min, the sections were stained by H&E Staining Kit Plus (MMI, Switzerland). The procedures were carried out according to the manufacturer’s instructions. The whole process was completed within 30 min [45].

### Laser capture microdissection

Microdissection was performed on a laser micro-cutting instrument (MMI, Switzerland), and the whole process was controlled in 1 h. The targeted cells on the slide were identified under microscope. Three important parameters of laser (cell velocity, laser focus, and laser power) were optimized. After circling the interesting area, the laser starts to capture the cells as many as possible. Finally, the LCM caps containing the captured tissue were uploaded and add 50 μl TPK Lysis Buffer (Micro Elute® RNA Kit, Omega, USA). RNA was extracted immediately or the sample was stored at − 80 °C (< 2 days).

### Library preparation for transcriptome sequencing

RNA were extracted using Micro Elute® RNA Kit (Omega, USA). A total amount of 1.5 μg RNA per sample was used for library preparation. NEBNext® Ultra™ RNA Library Prep Kit were used to generate sequencing libraries for Illumina® (NEB, USA) following the manufacturer’s recommendations. Index codes were added to attribute sequences to each sample. Then 3 μl USER Enzyme (NEB, USA) was used with size-selected, adaptor-ligated cDNA at 37 °C for 15 min followed by 5 min at 95 °C before PCR. PCR was performed with Phusion High-Fidelity DNA polymerase, Universal PCR primers, and Index (X) Primer. PCR products were purified (AMPure XP system) and library quality was assessed on the Bioanalyzer 2100 system (Agilent, USA). At last, the libraries were sequenced on an Illumina Hiseq platform and 150 bp paired-end reads were generated.

### Processing of raw reads and quantification of gene expression levels

Raw data (raw reads) of the fastQ format were first processed through in-house Perl scripts. In this step, clean data (clean reads) were obtained by removing reads containing adapter, reads containing ploy-N and low quality reads from raw data. At the same time, Q20, Q30, GC content and the clean data were calculated. Q20 indicates that every 100 bp of sequencing reads will have an error, and Q30 indicates that every 1000 bp of sequencing reads will have an error. All the downstream analyses were based on clean data with high quality. The clean reads were mapped to the orange-spotted grouper (*Epinephelus coioides*) genome (unpublished data). Index of the reference genome was built using Bowtie v2.2.3 and paired-end clean reads were aligned to the reference genome using TopHat v2.0.12. TopHat was selected as the mapping tool. HTSeq v0.6.1 was used to count the reads numbers mapped to each gene. And then FPKM of each gene was calculated based on the length of the gene and read counts mapped to this gene.

### DEGs analysis

Prior to DEGs analysis, for each sequenced library, the read counts were adjusted by edgeR program package through one scaling normalized factor. Differential expression analysis of two conditions was performed using the DEGSeq R package (1.20.0). The *P* values were adjusted using the Benjamini & Hochberg method. Corrected *P*-value of 0.05 and log 2 (fold change) of 1 were set as the threshold for significant differential expression.

### GO and KEGG enrichment analysis of differentially expressed genes

GO terms with corrected P value less than 0.05 were considered to be significantly enriched by DEGs. The identified DEGs were conducted for enrichment analysis subsequently by GO: Termfinder software using the hypergeometric test [46, 47], and *P*-values were corrected using the Bonferroni method [48]. Significantly enriched GO terms were selected by Q value (Q < 0.05). KEGG is a database resource for understanding high-level functions and utilities of the biological system from molecular-level information (http://www.genome.jp/kegg/). KOBAS software was used to test the statistical enrichment of differential expression genes in KEGG pathways (http://kobas.cbi.pku.edu.cn/m).

### Real-time PCR

To validate the RNA-seq data, the relative mRNA levels of 13 differentially expressed genes (*nr0b1*, *erα*, *erβ*, *wnt9*, *gdf9*, *bmp15*, *cyp17a1*, *hibadh*, *dhrs11*, *dhrs12*, *p5cdh*, *cyp3a40*, and *dhrs13*) were examined by quantitative real-time PCR in the gonad during spermatogenesis of orange-spotted grouper. Total RNA was extracted by TRIzol (Invitrogen, USA) and then 1 μg RNA from each sample was reverse transcribed with random primers by using the First Strand cDNA Synthesis Kit (Roche, USA) according to the manufacturer’s instruction. All mRNA quantification data were normalized to *ef1a* and presented as a relative control group. The specific primers used in this study were listed in Table 2.

**Table 2:** The primers used in real-time PCR

Primers	Purpose	Sequence (from 5’to 3′)
*cyp17a1*-F	real-time PCR	GGTATTACGGATCCGCCCTG
*cyp17a1*-R	real-time PCR	AGGTAGCTGGGTGATGGGAT
*3hd*-F	real-time PCR	GTACTGCGGTCAGGTTGGAA
*3hd*-R	real-time PCR	CTCCAGGCACAGGGTTGTAG
*sdr12*-F	real-time PCR	TAACAATGCTGGCTGTATGGTGAA
*sdr12*-R	real-time PCR	CTTCTTCAGTGCGGGTATCAGC
*sdr11*-F	real-time PCR	GAAAGACCGAGGGCTGGAG
*sdr11*-R	real-time PCR	CGATGCCCACCCATACTGTT
*p5cdh*-F	real-time PCR	AGCTGATGGTCCAGTGTTCG
*p5cdh*-R	real-time PCR	GTTCTTCCCTCCGCACTCTC
*cyp3A40*-F	real-time PCR	GCCTGAATGGAGACCTCTATGA
*cyp3A40*-R	real-time PCR	GGGGAAACACCCTTGGAACA
*sdr13*-F	real-time PCR	TGAGGCTGAATGGGAAGACG
*sdr13*-R	real-time PCR	CACACCTGCGTTGTTGATGAG
*nr0b1*-F	real-time PCR	CCAAGGAGTATGCGTATCTGAAA
*nr0b1*-R	real-time PCR	CGTTGAGAGCCTGGTGCG
*era*-F	real-time PCR	CTGCTCTCACACATCAGGCA
*era*-R	real-time PCR	TGATTTGTCAGGCCGTTGGA
*erb*-F	real-time PCR	CCACGGGACATTGCCTTAC
*erb*-R	real-time PCR	GCTCTTACGGCGGTTCTTGT
*wnt9*-F	real-time PCR	TGAGAACACCTGGCTCCAAC
*wnt9*-R	real-time PCR	CAGATGACTCTGACCAGGCG
*gdf9*-F	real-time PCR	CACAGTCAACGCAGAAAGGC
*gdf9*-R	real-time PCR	GACCAGAAACCTCTGGTGGG
*bmp15*-F	real-time PCR	ACCAGCACACCTAAGAACCG
*bmp15*-R	real-time PCR	GAATGGAAGCCGTAGGGGAG
*zbtb40*-Q-F	real-time PCR	ACCTTTGCTCACCCATCAGG
*zbtb40*-Q-R	real-time PCR	AATGGACTGGGCGAAGACAG
*zbtb40*-F3	semi-qPCR	TTTCACCAAACAGGGACGACT
*zbtb40*-R3	semi-qPCR	CCTCTGCGTCTTTACACCCATT

### Cloning and sequence analysis of *zbtb40* cDNA

Total RNA of the gonad was extracted by TRIzol (Invitrogen, USA). RNA was reversed to cDNA with First Strand cDNA Synthesis Kit (Roche, USA). Based on the cDNA fragments in RNA-seq data, specific upstream and downstream primers (Table 3) were designed. The primers were used to amplify the ORF of *zbtb40*. After PCR amplification, the band of the desired size was purified by the E.Z.N.A. Gel Extraction Kit (Omega, USA). The purified product was then subcloned into the pGEM-Easy vector (Fermentas, USA). According to the sequencing result, the ORF of *zbtb40* was obtained.

**Table 3:** The primers used in verifying the quality of samples

Primers	Purpose	Sequence (from 5’to 3′)
*ef1a*-F	verifying	GGTCGTCACCTTCGCTCCAT
*ef1a*-R	verifying	TCCCTTGGGTGGGTCATTCT
*dmrt1*-F	verifying	GCTGGAGTAGACTGCTTGTTT
*dmrt1*-R	verifying	CGACTGTGCGTCAGTATGAGC
*slbp2*-F	verifying	CGAAGATGACCTCCGACCTG
*slbp2*-R	verifying	CGGAGCCAGTCAGTCATGTT
*vasa*-F	verifying	ACCAGATCTTCCTGGAGGC
*vasa*-R	verifying	CAAATGACTGCTCCACGTCA
*zbtb16*-F	verifying	GCAGGGGACCATCCATTTGA
*zbtb16*-R	verifying	CACACGGTAGTGGGTTTCCA
*zbtb40*-F1	verifying	TTTGCTCACCCATCAGGCAT
*zbtb40*-R1	verifying	CCAAAGAGGCGAAGCTGAGA
*zbtb40*-F2	ISH	TCTCAGCTTCGCCTCTTTGG
*zbtb40*-R2	ISH	AGAGATCACTGACTCCGCCT

The putative amino acid sequences were predicted by DNAMAN software. Nucleic acid phylogenetic analysis was conducted with MEGAX using the method of neighbor-joining method and the top ten motif sites were predicted by motif-based sequence analysis tools (MEME, http://meme-suite.org/).

### Tissue distribution of *zbtb40* and its expression profile MT-induced spermatogenesis

Total RNA from eight tissues (whole brain, heart, head kidney, liver, kidney, pituitary, ovary, and testis) was extracted. RNA was reversed to cDNA with First Strand cDNA Synthesis Kit (Roche, USA). The reverse transcription process was as follow: 37 °C for 15 min, 98 °C for 5 min, and 4 °C for 5 min. The PCR amplification regime was 35 cycles of 94 °C for 20 s, 55 °C for 10 s, and 72 °C for 20 s, followed by a further amplification at 72 °C for 5 min.

The expression profile of *zbtb40* in the gonad during spermatogenesis was detected by real-time PCR during MT-induced spermatogenesis. The methods were carried out as described above. The specific primers used in this study were listed in Table 3.

### In situ localization of *zbtb40* in gonads during MT-induced spermatogenesis

The protocol of in situ localization (ISH) was referred to previous papers with minor modifications [49]. Probes of *zbtb40* (628 bp) were synthesized by RNA DIG Labeling Kit (Roche, USA) according to the manufacturer’s instructions. After being permeabilized and acetylated, the cryosections were incubated by hybridization solution which contained 1 μg/ml probe, 20 x saline solution citrate (SSC) buffer, salmon sperm DNA, and deionized formamide, 50 x Denhart’s solution. After incubation for 12–16 h, the sections were washed by SSC and PBS buffer. The DIG label was tested with an alkaline phosphatase conjugated Flu-anti-DIG antibody (Roche Diagnostics; diluted 1:1000) and colored the signal with Fluorescence Systems (Roche, USA). Later the sections were counterstained by 4′ 6-diamidino-2-phenylindole (DAPI) for cell nuclear staining to confirm the number and status of germ cell. At last, sections were mounted with the Gold Anti-fade reagent (Invitrogen, USA) and imaged by laser scanning confocal microscope (Leica, TCS-SP5, Germany).

### Dual-label in situ hybridization of *zbtb40* and *cyp17a1* in gonads

The protocol of dual-label in situ hybridization was referred to previous study with minor modifications [50]. Briefly, the sample was hybridized by DIG-labeled *zbtb40* RNA probes and biotin-labeled *cyp17a1* RNA probes at the same time in 58 °C oven. After hybridization, the sections were washed by SSC and PBS buffer. The DIG label was tested with an alkaline phosphatase conjugated Flu-anti-DIG antibody, and the biotin-label was tested with an alkaline phosphatase conjugated streptavidin-POD antibody. The positive signals of ISH were developed by the TSA Plus Fluorescence Systems according to the product manual (Roche, USA). The signals of *zbtb40* were red, and the signals of *cyp17a1* were green. Cell nuclei were stained by DAPI (blue). Finally, the sections were mounted with the Gold Anti-fade reagent (Invitrogen, USA) and imaged with a microscope (Leica, TCS-SP5, Germany).

### Statistical analysis

All data were expressed as mean values ± SEM. Significant differences were checked by one-way analysis of variance (ANOVA) and student′s t-test was used, and a probability level less than 0.05 (*P* < 0.05) was used to indicate significance. All data were performed using GraphPad Prism5.0 (GraphPad Software, San Diego, CA) and analyzed by SPSS17.0 (SPSS, Chicago, IL, USA).

## Supplementary information

**Additional file 1 : Figure S1.** GO classification analysis of all DEGs. **Figure S2.** Validation of selected genes using real-time PCR during sex reversal. **Figure S3.** Validation of *zbtb* genes using RT-PCR. **Figure S4.** The nucleotide sequences and deduced amino acid sequences of *zbtb40*.

**Additional file 2 : Table S1.** The detailes of the top 20 KEGG enriched pathway. **Table S2.** The top 10 conserved sites among 14 species.

## Data Availability

The datasets used and analyzed in the current study are available from the corresponding author on reasonable request. All relevant data are within the paper and the transcriptome data can be obtained from the Transcriptome Shotgun Assembly project DDBJ under accession number SSUB012433.

## Abbreviations

DEPC: Diethyl pyrocarbonate
DIG: Digoxigenin
GO: Gene Ontology
KEGG: Kyoto encyclopedia of genes and genomes database
PBS: Phosphate buffered solution
PEN: Polyethylene naphthalate
