# Supplementary material for: Transcriptome profiling of laser-captured germ cells and functional characterization of zbtb40 during 17alpha-methyltestosterone-induced spermatogenesis in orange-spotted grouper (Epinephelus coioides)
Source: BMC Genomics. 2020 Jan 23;21:73. doi: 10.1186/s12864-020-6477-4 (PMC6979330; doi:10.1186/s12864-020-6477-4)
Supplement: Supplementary file 1 — Additional file 1 : Figure S1. GO classification analysis of all DEGs. Figure S2. Validation of selected genes using real-time PCR during sex reversal. Figure S3. Validation of zbtb genes using RT-PCR. Figure S4. The nucleotide sequences and deduced amino acid sequences of zbtb40. [file 12864_2020_6477_MOESM1_ESM.docx]

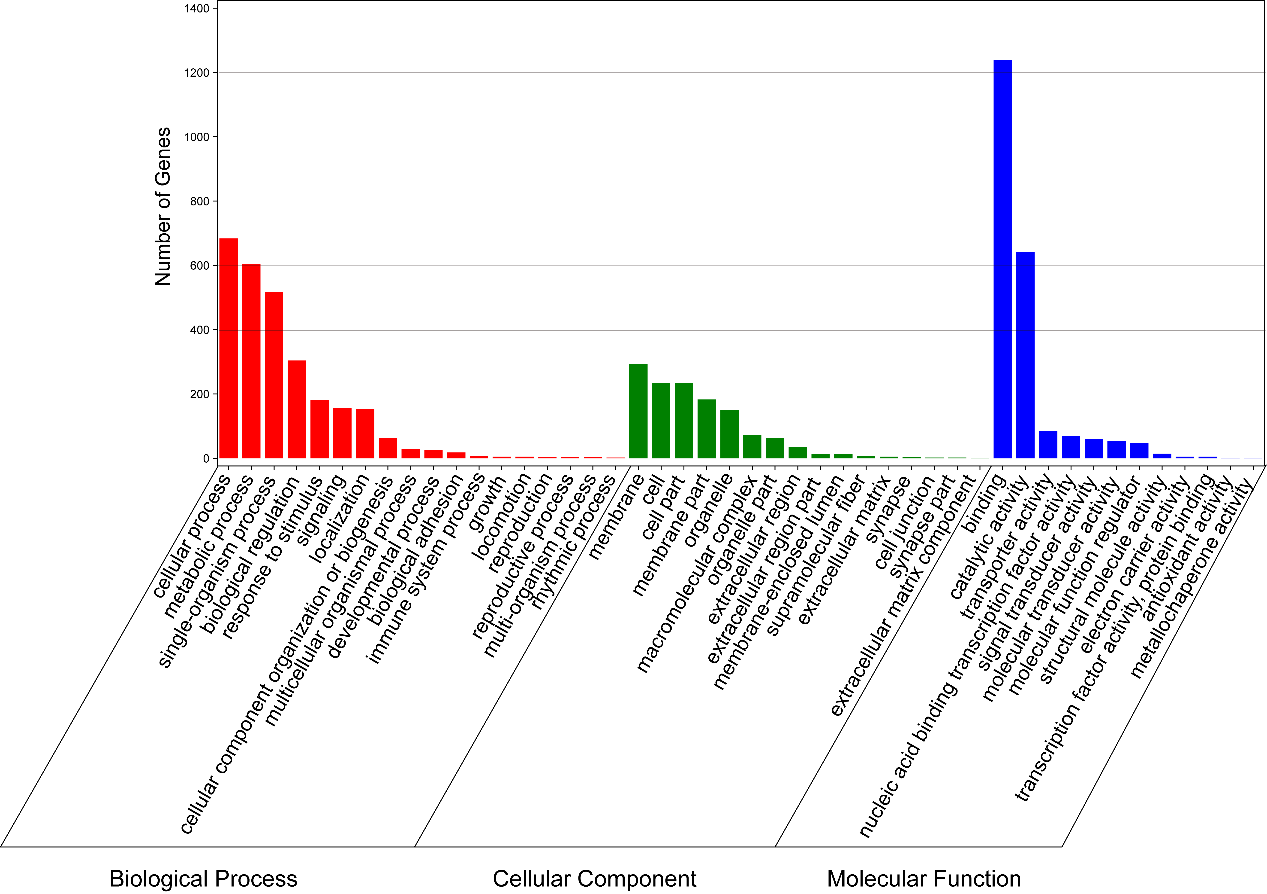


Fig. S1. GO classification analysis of all DEGs. GO functions are shown in X axis. The Y axis shows the numbers of genes which have the GO function.

Fig. S2. Validation of selected genes using real-time PCR during sex reversal. **(A)** *hibadh*, **(B)** *dhrs11*, **(C)** *dhrs12*, **(D)** *p5cdh*, **(E)** *cyp3a40*, **(F)** *dhrs13*.


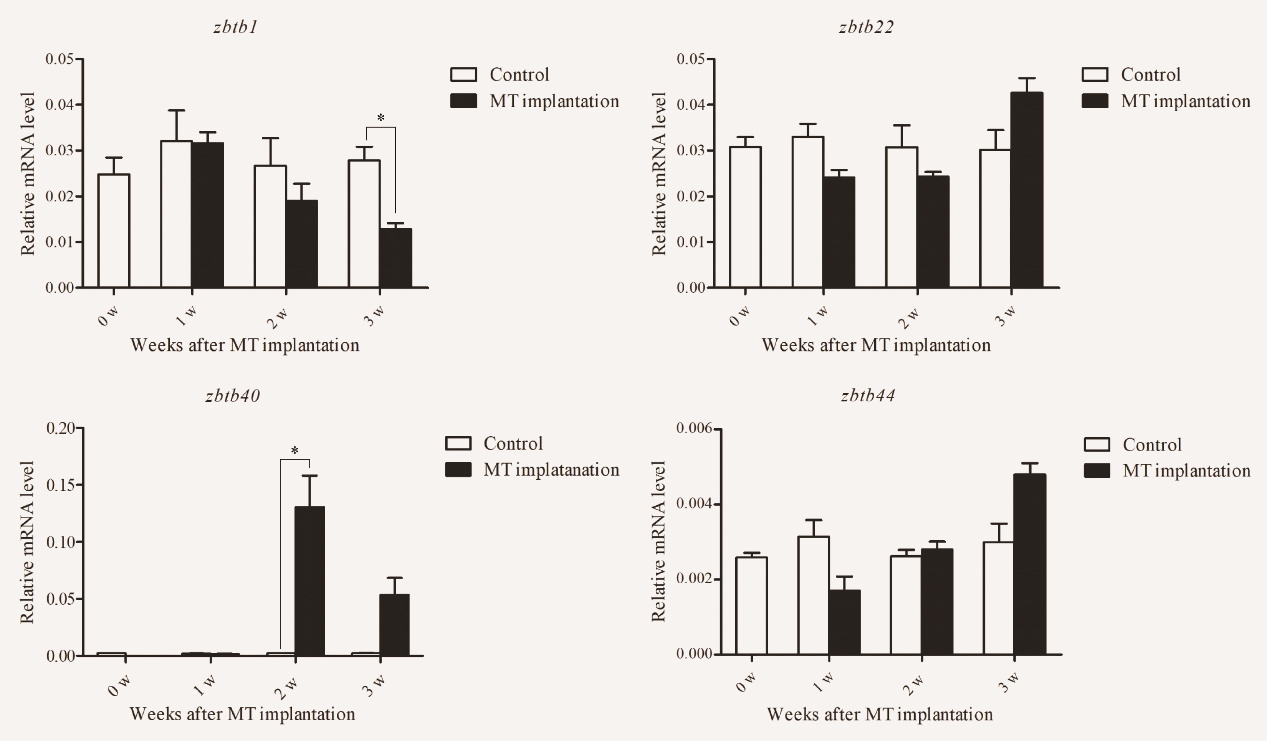


Fig. S3. Validation of *zbtb* genes using RT-PCR. (A) *zbtb1*, (B) *zbtb22*, (C) *zbtb40*, (D) *zbtb44*.


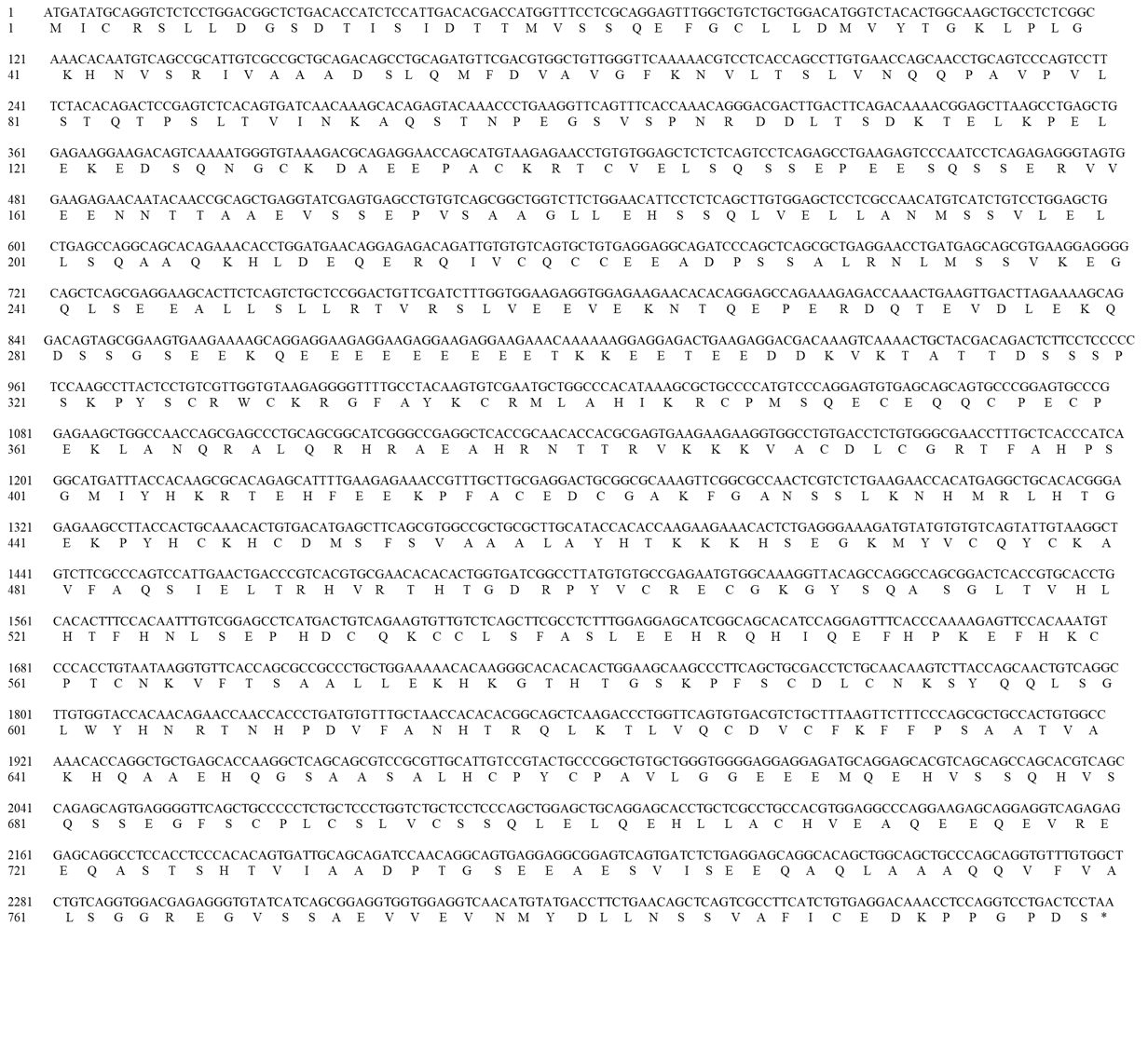


Fig. S4. The nucleotide sequences and deduced amino acid sequences of *zbtb40*. The stop codon was denoted by an asterisk. Nucleotide and amino acid were numbered on the left.
